# Urinary polycyclic aromatic hydrocarbon metabolites and adult asthma: a case-control study

**DOI:** 10.1038/s41598-018-26021-3

**Published:** 2018-05-16

**Authors:** Xiji Huang, Yun Zhou, Xiuqing Cui, Xiaojie Wu, Jing Yuan, Jungang Xie, Weihong Chen

**Affiliations:** 1 Department of Occupational & Environmental Health and Key Laboratory of Environment and Health, Ministry of Education & Ministry of Environmental Protection, and State Key Laboratory of Environmental Health (Incubating), School of Public Health, Tongji Medical College, Huazhong University of Science and Technology, Wuhan, 430030 China; 2 Department of Respiratory and Critical Care Medicine, Tongji Hospital, Tongji Medical College, Huazhong University of Science and Technology, Wuhan, 430030 China; 3 Hubei Provincial Center for Disease Control and Prevention, Wuhan, Hubei China

## Abstract

Polycyclic aromatic hydrocarbons (PAHs) exposure was reported to be associated with childhood asthma. However, the quantitative relationship between PAHs exposure and adult asthma and possible inflammatory pathways are less clear. We aimed to investigate potential associations between urinary PAHs metabolites and adult asthma. We enrolled 507 adult asthma cases and 536 matched controls. The concentrations of 12 urinary PAHs metabolites and plasma cytokines of interleukin (IL)-9 and eotaxin were measured. Potential associations between urinary PAHs metabolites and adult asthma were analyzed by logistic regression. The relationships between urinary PAHs metabolites and plasma cytokines were determined by generalized linear regression. After adjusted for covariates, each 1-unit-increase in natural log-transformed concentrations of 2-hydroxyfluorene (2-OHFLU), 4- hydroxyphenanthrene (4-OHPHE), 1-OHPHE, 2-OHPHE, 1-Hydroxypyrene (1-OHPYR) and ∑OH-PAHs were significantly associated with elevated risk of adult asthma with odds ratios of 2.04, 2.38, 2.04, 1.26, 2.35 and 1.34, respectively. And the associations were more pronounced in the subjects who were female, younger than 45 years, smoker and had history of occupational dust exposure. No associations were observed between urinary PAHs metabolites levels and expressions of IL-9 and eotaxin. Our results demonstrated that elevated urinary PAHs metabolites levels were associated with increased risk of asthma in adults.

## Introduction

Asthma is one of the most common chronic inflammatory lung diseases in Asia, like other continents^1^. It is characterized by recurrent symptoms of airway obstruction, coughing and wheezing. Nowadays, the prevalence of asthma has continuously increased, and approximately 300 million individuals suffered from asthma worldwide. In China, the asthmatic population has reached 30 million, and the prevalence of asthma in higher developed cities like Beijing or Shanghai has increased by over 150% in the last ten years^2^.

Growing literature both from epidemiological work as well as experimental studies suggest that indoor and outdoor air pollution like particulate matter, trace metals and polycyclic aromatic hydrocarbons (PAHs) contribute to the development of allergy and asthma^3^. PAHs are a group of hydrocarbons defined by two or more fused aromatic rings produced during the incomplete combustion of organic material such as fossil fuels, coal, wood, and tobacco. Individuals are exposed to PAHs through inhalation, ingestion or percutaneous penetration. In the past, most of studies reported the qualitative associations between exposure to PAHs in the ambient air and asthma. Only several studies focused on the quantitative association between PAHs exposure and childhood asthma, and results were inconsistent. Several studies supported that exposure to PAHs increased the risk of asthma in childhood^4,5^. Nevertheless, Rosa found that not only prenatal but also postnatal PAHs exposure was not associated with asthma in children aged 5–6 years, and even a negative nonsignificant association was found between prenatal PAHs exposure and asthma among children without prenatal environmental tobacco smoking exposure^6^. To our knowledge, there is no quantitative study on the association of exposure to PAHs with adult asthma which is partly different from childhood asthma in clinical, biological and genetic characteristics^7^. Additionally, most of previous studies applied the data of air monitoring to evaluate the personal exposure to PAHs. Given that diet is another major source of PAHs exposure of the general population and majority of PAHs are excreted in urine, the obtained data on urine concentrations of PAHs metabolites reflect a more accurate estimation of the quantity of the actual personal PAHs intake compared to ambient air measurements^8^.

In addition, airway inflammatory responses were fundamental to asthma pathogenesis. Studies suggest that PAHs may influence the development of asthma through inflammatory responses. Experimental studies showed that exposure to PAHs might influence B cell and T-helper cell differentiation by skewing immune responses toward a Th2 specific profile, which favored B-cell production of immunoglobulin E (IgE) and eosinophils^9^. As important Th2 cytokines, interleukin (IL)-9 and eotaxin are reported to be involved in the development of asthma, and are supposed to be useful in the diagnosis and assessment of asthma^10,11^. In our previous study, the results from both high-throughout protein chip and enzyme-linked immunosorbent assay (ELISA) kits showed that the expressions of IL-9 and eotaxin were different between asthma cases and healthy controls^12^. Hence, we hypothesized that levels of PAHs exposure might be associated with asthma through changing the expression levels of IL-9 and eotaxin.

In the present study, we determined and compared the levels of PAHs metabolites in urine in asthma cases and matched controls. The objective was to quantify the dose-response relationships between urinary PAHs metabolites and adult asthma, and analyze the potential associations between urinary PAHs metabolites and expression of plasma IL-9 and eotaxin.

## Results

### General characteristics

A total of 507 asthma cases and 536 matched controls were included in the final analysis. The comparison of general characteristics of subjects is presented in Table 1. Male subjects accounted for 43.79% in cases and 43.28% in controls. Mean age was 42.09 ± 12.89 years for asthma cases and 42.76 ± 12.51 years for controls. The distributions of educational level, tobacco smoking, alcohol consumption, physical activity and flower gardening were significantly different between asthma cases and controls (all *P* < 0.05). Of note, positive history of asthma, pet ownership and occupational dust exposure were more prevalent in the asthma cases than in controls (*P* < 0.0001, *P* = 0.0016 and *P* = 0.0279, respectively). Compared with controls, each index of spirometry was significantly higher in asthma cases (all *P* < 0.01). The expression of IL-9 in asthma cases was significant higher than that in controls, but the expression of eotaxin was significant higher in controls when compared with cases.

**Table 1:** General characteristics of asthma cases and controls.

Characteristic	Controls (n = 536)	Cases (n = 507)	*p*-value
Male, *n* (%)	232 (43.28)	222 (43.79)	0.8698
Age (years, Mean ± SD)	42.76 ± 12.51	42.09 ± 12.89	0.6818
Educational level, *n* (%)			0.0006
Low	253 (43.20)	283 (55.82)	
Middle	193 (36.01)	127 (25.05)	
High	90 (16.79)	97 (19.13)	
Exposure to dust, *n* (%)	68 (12.69)	89 (17.55)	0.0279
Family history of asthma, *n* (%)	11 (2.05)	53 (10.45)	<0.0001
Tobacco smoking, *n* (%)			<0.0001
Nonsmokers	380 (70.90)	399 (78.70)	
Former smokers	27 (5.04)	39 (7.69)	
Current smokers	129 (24.07)	69 (13.61)	
Alcohol consumption, *n* (%)			<0.0001
Nondrinkers	401 (74.81)	430 (84.81)	
Former drinkers	16 (2.99)	28 (5.52)	
Current drinkers	119 (22.20)	49 (9.66)	
Physical activity, *n* (%)	184 (34.33)	121 (23.87)	0.0002
Pet ownership, *n* (%)	56 (10.45)	87 (17.16)	0.0016
Flower gardening, *n* (%)	166 (30.97)	103 (20.32)	<0.0001
BMI (kg/m^2^, Mean ± SD)	24.01 ± 3.63	23.12 ± 4.28	0.0004
Spirometric indices (Mean ± SD)			
FEV_1_ (L)	2.49 ± 0.73	2.32 ± 0.86	0.0009
FEV_1_ (% predicted)	85.59 ± 19.92	81.43 ± 24.76	0.0029
FEV_1_/FVC (%)	85.91 ± 15.65	71.60 ± 14.14	<0.0001
Cytokines (Mean ± SE)			
IL-9 (pg/ml)	35.47 ± 6.70	71.79 ± 7.45	0.0028
Eotaxin (pg/ml)	57.73 ± 2.52	47.50 ± 2.52	<0.0001

Abbreviation: SD, standard deviation; SE, standard error; BMI, body mass index; FEV_1_, forced expiratory volume in 1 s; FVC, forced vital capacity.

Levels of urinary PAHs metabolites are showed in Table 2. Levels of 2-OHFLU, 4-OHPHE, 1-OHPHE, 2-OHPHE, 1-OHPYR and ∑OH-PAHs were significantly higher in asthma cases than those in controls (all *P* < 0.01). No significant differences in 1-OHNAP, 2-OHNAP, 9-OHFLU, 9-OHPHE and 3-OHPHE were observed between asthma cases and controls.

**Table 2:** Levels of urinary PAHs metabolites (μg/mmol creatine) in asthma cases and controls.

PAHs metabolites	Controls (n = 536)	Cases (n = 507)	*P*-value
1-OHNAP	0.54 (0.34–0.80)	0.56 (0.37–0.74)	0.8942
2-OHNAP	1.16 (0.70–1.74)	1.11 (0.75–1.38)	0.0890
9-OHFLU	0.84 (0.46–1.48)	0.84 (0.49–1.24)	0.7349
2-OHFLU	0.26 (0.17–0.35)	0.32 (0.22–0.39)	<0.0001
4-OHPHE	0.28 (0.18–0.41)	0.36 (0.27–0.43)	<0.0001
9-OHPHE	0.60 (0.40–0.89)	0.64 (0.42–0.85)	0.3586
3-OHPHE	0.30 (0.20–0.45)	0.31 (0.18–0.47)	0.6072
1-OHPHE	0.22 (0.10–0.23)	0.27 (0.09–0.36)	<0.0001
2-OHPHE	0.16 (0.14–0.30)	0.19 (0.18–0.37)	0.0028
1-OHPYR	0.68 (0.42–1.09)	0.91 (0.69–1.06)	<0.0001
∑OH-PAHs	5.55 (3.78–7.57)	6.06 (4.24–7.64)	0.0388

Values are presented as geometric mean (25th–75th quartiles).

### Associations between asthma and PAHs metabolites

Table 3 shows the odds ratios (ORs) and 95% confidential interval (CI) for asthma by logistic regression. After adjusted by gender, age, educational level, occupational dust exposure, family history of asthma, tobacco smoking, alcohol consumption, pet ownership, flower gardening, physical activity, and BMI, we observed that each 1-unit-increase s in the natural log-transformed concentrations of 2-OHFLU, 4-OHPHE, 1-OHPHE, 2-OHPHE, 1-OHPYR and ∑OH-PAHs was significantly associated with elevated risk of asthma with ORs of 2.04, 2.38, 2.04, 1.26, 2.35 and 1.34, respectively. When concentrations of PAHs metabolites were divided into quartiles, the increased levels of 2-OHFLU, 4-OHPHE, 1-OHPHE, 2-OHPHE, and 1-OHPYR were significantly positively associated with asthma. Compared with the first quartile, the adjusted ORs of asthma gradually increased and slightly declined in the other quartiles in 2-OHFLU, 4-OHPHE, 1-OHPHE and 1- OHPYR, except in 2-OHPHE. It is worth noting that the second and third quartile of 1-OHPYR were associated with >15 times higher risk of asthma compared with the first quartile. In addition, we did not observe significant associations of asthma with 1-OHNAP, 2-OHNAP, 9-OHFLU, 9-OHPHE and 3-OHPHE in both models.

**Table 3:** Odds ratio and 95% confidential interval for asthma by considering urinary PAHs metabolites as continuous or categorical variables.

PAHs metabolites	as continuous variables	*P*-value	as categorical variables				
			quartile 1	quartile 2	quartile 3	quartile 4	*P*-value for trend
1-OHNAP	1.13 (0.91–1.41)	0.2752	1 (ref)	1.97 (1.35–2.88)	1.45 (0.99–2.12)	1.05 (0.71–1.54)	0.8090
2-OHNAP	0.90 (0.72–1.11)	0.3179	1 (ref)	2.48 (1.69–3.65)	1.26 (0.86–1.85)	0.87 (0.59–1.29)	0.0770
9-OHFLU	0.91 (0.76–1.08)	0.2675	1 (ref)	1.42 (0.97–2.06)	1.04 (0.71–1.53)	0.77 (0.52–1.14)	0.0787
2-OHFLU	2.04 (1.58–2.64)	<0.0001	1 (ref)	2.58 (1.75–3.82)	3.32 (2.24–4.91)	2.73 (1.84–4.07)	<0.0001
4-OHPHE	2.38 (1.82–3.11)	<0.0001	1 (ref)	5.31 (3.47–8.11)	7.20 (4.76–10.89)	4.37 (2.87–6.65)	<0.0001
9-OHPHE	1.14 (0.92–1.42)	0.2227	1 (ref)	0.99 (0.68–1.44)	1.45 (0.99–2.12)	0.99 (0.68–1.47)	0.5289
3-OHPHE	1.06 (0.89–1.26)	0.5367	1 (ref)	0.62 (0.42–0.90)	0.88 (0.60–1.29)	0.89 (0.61–1.30)	0.9959
1-OHPHE	2.04 (1.60–2.61)	<0.0001	1 (ref)	1.69 (1.15–2.50)	3.74 (1.85–4.04)	3.14 (2.08–4.74)	<0.0001
2-OHPHE	1.26 (1.08–1.47)	0.0033	1 (ref)	0.32 (0.21–0.47)	0.61 (0.42–0.89)	1.42 (0.97–2.07)	0.0081
1-OHPYR	2.35 (1.85–3.00)	<0.0001	1 (ref)	15.55 (9.49–25.49)	15.42 (0.39–25.32)	7.43 (4.53–12.18)	<0.0001
∑OH-PAHs	1.34 (1.03–1.74)	0.0278	1 (ref)	1.93 (1.32–2.83)	1.32 (0.90–1.93)	1.37 (0.93–2.04)	0.3906

Models were adjusted by gender, age, educational level, occupational dust exposure, family history of asthma, tobacco smoking, alcohol consumption, pet ownership, flower gardening, physical activity, and body mass index.

We also performed the same analyses for the risk of urinary PAHs metabolites for asthma in exacerbation and remission. The natural log-transformed concentrations of 2-OHFLU, 4-OHPHE, 1-OHPHE, 2-OHPHE, 1-OHPYR and ∑OH-PAHs were positively associated with asthma in exacerbation, as well as asthma in remission. The risk of asthma also tended to increase at low exposure levels of PAHs metabolites but attenuate or even decline at high exposure levels.

### Stratified analyses for associations between PAHs metabolites and asthma

**Figure 1:**
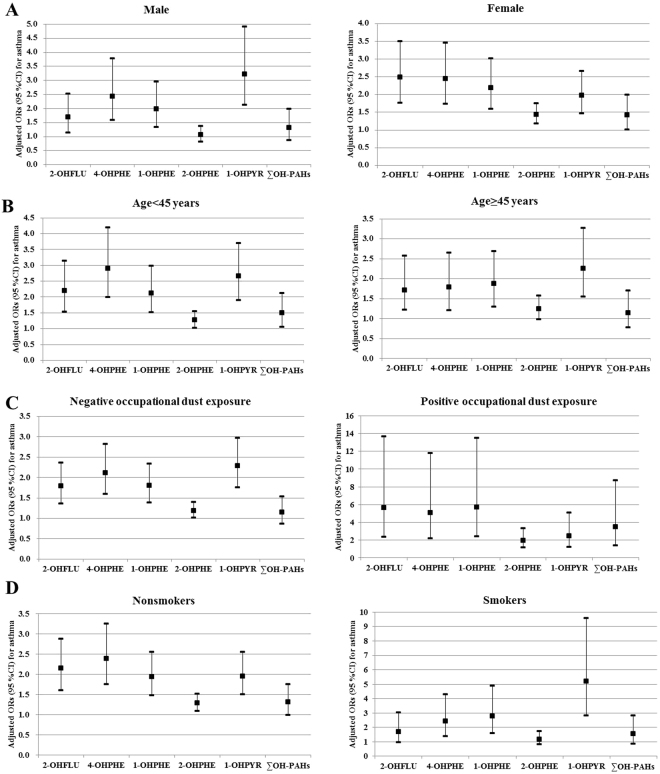
Adjusted odds ratios for asthma according to quartiles of creatinine-corrected OH-PAHs stratified by gender (**A**), age (**B**), occupational dust exposure (**C**) and tobacco smoking (**D**). All covariates were gender, age, educational level, occupational dust exposure, family history of asthma, tobacco smoking, alcohol consumption, pet ownership, flower gardening, physical activity, and BMI. Each group adjusted for the other covariates except itself.

We further stratified the subjects by main characteristics including gender, age, occupational dust exposure and tobacco smoking, and analyze the associations of asthma with PAHs metabolites (2-OHFLU, 4-OHPHE, 1-OHPHE, 2-OHPHE, 1- OHPYR and ∑OH-PAHs) in different subgroups (Fig. 1). In all, we observed the significant dose-response trends between the selected PAHs metabolites and increased odds of asthma in the subjects who were female, younger than 45 years, smoker and had history of occupational dust exposure. Additionally, we found the significant interactions on risk of asthma between the total urinary PAHs metabolites and certain characteristics including gender, age and occupational dust exposure.

### Association between PAHs and cytokines

We further analyzed the association between urinary PAHs metabolites and expression of plasma cytokines (Table 4). Among the selected asthma-related PAHs metabolites (2-OHFLU, 4-OHPHE, 1-OHPHE, 2-OHPHE, 1- OHPYR and ∑OH-PAHs), we did not find any metabolites were significantly associated with the expressions of IL-9 and eotaxin, after adjusted for gender, age, occupational dust exposure, tobacco smoking, BMI.

**Table 4:** Associations between serum cytokines and PAHs metabolites.

PAHs metabolites	IL-9		Eotaxin	
	β	*P*-value	β	*P*-value
2-OHFLU	0.04	0.7061	0.07	0.1537
4-OHPHE	0.13	0.3137	−0.01	0.8995
1-OHPHE	0.08	0.4759	0.08	0.0839
2-OHPHE	−0.07	0.2551	0.05	0.0683
1-OHPRY	0.14	0.2363	−0.02	0.6363
∑OH-PAHs	−0.03	0.8318	0.09	0.0808

The model were adjusted for gender, age, occupational dust exposure, tobacco smoking, BMI.

## Discussion

In the present study, we found potential associations between certain urinary PAHs metabolites and risk of adult asthma. After adjustment for confounders, we observed that elevated risk of asthma in adults was significantly associated with the increases in concentrations of urinary 2-OHFLU, 4-OHPHE, 1-OHPHE, 2-OHPHE, 1-OHPYR, as well as the total urinary PAHs metabolites. No significant associations between urinary PAHs metabolites and expressions of IL-9 and eotaxin were observed.

Because asthma is the most common chronic disease in children, previous studies about the effect of PAHs exposure on asthma were mostly conducted in children. In a case-control study in India, the levels of phenanthrene in asthma cases aged 1–14 years is over 15 times higher than that in controls^13^. The Fresno Asthmatic Children’s Environment Study (FACES) also reported that phenanthrene had a higher relative impact on wheeze^4^. However, the Columbia Center for Children’s Environmental Health birth cohort (CCCEH) found that PAHs exposure without environmental tobacco smoking or obesity was not directly associated with asthma or wheeze in childhood^6,14,15^, and only repeated high exposure to pyrene was associated with asthma^5^. The research group of CCCEH also found no association of urinary PAHs metabolites with childhood asthma^16^, except when children were repeatedly exposed to high levels of PAHs^17^. Compared with childhood asthma, adult asthma may be more contributed to occupational or environmental irritant exposure^18^. In the present study, we observed dose-response relationships between urinary PAHs metabolites and asthma in adults. To our knowledge, no study has investigated the quantitative relationship between urinary PAHs metabolites with adult asthma, especially in China. These findings have important implications in public health.

We also determined the levels of IL-9 and eotaxin, which were considered to be helpful in the diagnosis of asthma. We found that the average levels of IL-9 in asthma cases (71.79 ± 7.45 pg/ml) were significantly higher than that in controls (35.47 ± 6.70 pg/ml). This was consistent with the previous study (cases vs. controls: 63.37 ± 9.27 pg/ml vs. 30.23 ± 1.92 pg/ml)^10^. The prior study reported higher levels of eotaxin in asthma cases^19^, but we observed the lower expressions of eotaxin in asthma cases than in controls in this study. Our findings were partly supported by Dent’s study, which did not find difference in sputum eotaxin between mild asthma cases and healthy controls^20^. Moreover, Schnyder-Candrian *et al*. reported that in asthma model mice, IL-17 was overproduced and subsequently largely inhibited the expression of chemokines including eotaxin, which may partly explain our finding^21^.

To date, the mechanism by which PAHs influence the development of asthma is still less clear. Studies suggest that PAHs may act through IgE to stimulate inflammatory responses and enhance allergic reactions. Experimental evidence indicates that pyrene enhances allergic IgE response in mice^22^. A case-control study in Saudi also found positive associations between serum PAHs levels and expressions of IgE and IL-4^23^. However, some other studies failed to support it. Rosa *et al*. reported the negative association between prenatal PAHs exposure and IgE^6^. Jung *et al*. only observed the association between PAHs exposure with asthma in nonatopic children whose specific IgE levels less than 0.35 IU/mL^5^. In this study, we did not observe the significant associations between PAHs exposure with levels of IL-9 and eotaxin. In addition, PAHs exposure is known to promote the production of reactive oxygen species and induce oxidative stress. Oxidative stress plays an important role in asthma. Hence, PAHs exposure has been linked to asthma through oxidative stress pathways. In coke oven workers who exposed to high levels of PAHs, higher malondialdehyde levels were observed^24^. Li *et al*. also reported that PAHs in diesel exhaust can initiate a cascade of oxidative stress that leads to airway inflammation^25^. Despite all this, the mechanism still need more research.

Furthermore, we found that the association between in the subjects who were female, younger than 45 years, smoker and had history of occupational dust exposure. As the source of PAHs, tobacco smoking and occupational dust exposure have been reported to be the risk factors of asthma^6,15,26^. In agreement with our study, Guo *et al*. also found that women were more susceptible than men to PAHs induced oxidative stress^27^. The pronounced PAHs-asthma association in women is likely due to gender-related differences in the metabolism of PAHs^28^. Absorbed PAHs are metabolized by cytochrome P450 (CYP) enzymes especially CYP1A1, to form epoxides. Compared with men, women display higher levels of BaP-DNA adducts and CYP1A1 gene expression in lungs when exposed to BaP^29^. Regarding age, we cannot explain the difference in the odds of asthma, but another study also found the PAHs-diabetes association was more prominent in individuals <55 years than those older than 55 years^30^.

There were several limitations in our study. First, spot urine samples were used for analysis which may result in measurement error due to individual variability in short-term excretion. Second, we only measured 12 PAHs metabolites, but other PAHs metabolites or environmental chemicals like POPs were not determined. Third, except some important confounders, other covariates like dietary factors were not included in the adjustment. Forth, although we observed differences in urinary PAHs metabolites levels between asthma cases and controls, it remains difficult to establish a causal relationship.

## Conclusion

In the present study, we observed the dose-responsive relationships between elevated urinary PAHs metabolites levels and increased risk of asthma in adults. PAHs-asthma association was more prominent in subjects who were female, younger than 45 years, smoker and had history of occupational dust exposure. The expressions of plasma cytokines IL-9 and eotaxin were not associated with levels of urinary PAHs metabolites. Prospective research is needed to confirm the causal relationship between PAHs exposure and adult asthma and explore the mechanisms involved.

## Methods

### Study population

From October 2010 through January 2012, we recruited 551 adult asthma cases from a general hospital in Wuhan, China. The cases were diagnosed by qualified physicians if they had symptoms such as recurrent breathlessness, wheezing, cough, and chest tightness according to the Global Initiative for Asthma (GINA) guidelines, and/or spirometry demonstrating an increase in forced expiratory volume in 1 s (FEV_1_) of at least 12% and at least 200 ml from the prebronchodilator value^31,32^. The controls without any respiratory problems were collected from the same residential areas where the asthma cases lived and matched with asthma cases for age and gender. We excluded the subjects among asthma cases and controls if they had any known infection, heart failure, and complications of other diseases, such as hypertension, diabetes and cerebral vessel disease at the time of study. After exclusion, 507 adult asthma cases and 536 controls were remained. According to the clinical symptoms, asthma cases were divided into acute exacerbation and clinical remission^33^. The numbers of asthma cases in acute exacerbation and clinical remission stage were 321 (63.3%) and 186 (36.7%), respectively.

Data on demographic information, occupational history and life style were collected by trained investigators during face-to-face interviews using uniform questionnaires. For asthma cases, the basic information before and after the latest asthma attack were obtained and nearly unchanged. Educational level was defined as low (below senior high school), middle (senior high school to technical school), or high (college degree or beyond). Individuals who had smoked more than one cigarette per day over the previous 6 months were considered as current smokers. Individuals who had drunk alcohol beverage more than once a week over the previous 6 months were considered as current drinkers. Physical activity was defined as regular exercise of more than 30 minutes in each session, at least once a week during previous six months.

Height and weight were measured and body mass index (BMI) was calculated as an index of physique. Spirometry were performed for all the subjects using a portable spirometer (HI-101, Chestgraphy, Japan) according to the American Thoracic Society/European Respiratory Society (ATS/ERS) guidelines^34^. Forced vital capacity (FVC) and FEV_1_ were obtained.

The study was approved by the Ethnics and Human Subject Committees of Tongji Medical School at the Huazhong University of Science and Technology. Written informed consents were obtained from all participants. All experiments were performed in accordance with the approved guidelines and regulations.

### Determination of PAHs metabolites and creatinine

Morning urine samples of all the subjects were collected and frozen in aliquots at −20 °C. We measured 12 PAHs metabolites in 3 ml urine samples using the Agilent 5975B/6890 N GC-MS System (Agilent, Santa Clara, CA, USA). Twelve PAHs metabolites are listed by the peak times: 1-hydroxynaphthalene (1-OHNAP), 2-OHNAP, 9-hydroxyfluorene (9-OHFLU), 2-OHFLU, 4-hydroxyphenanthrene (4-OHPHE), 9-OHPHE, 3-OHPHE, 1-OHPHE, 2-OHPHE, 1-Hydroxypyrene (1-OHPYR), 6-hydroxychrysene (6-OHCHR), and 3-hydroxybenzo[a]pyrene (3-OHBaP). The methods for determination have been previously published^35^. The limits of detection (LOD) for urinary PAHs metabolites range from 0.1 to 0.9 μg/L. For levels of PAHs metabolites below the LOD, we assigned half the LOD for calculation. Because 6-OHCHR and 3-OHBaP were below the limits of quantification, we only analyzed 10 PAHs metabolites, and ∑OH-PAHs represented the summation of the ten metabolites. We measured urinary creatinine concentrations using a fully automated clinical chemistry analyzer (Mindray Medical International Ltd., Shenzhen, China). The concentrations of PAHs metabolites were adjusted by creatinine and presented as micrograms per millimole of creatinine. Because the creatinine-adjusted PAHs metabolites concentrations had a positively skewed distribution, a natural log-transformation was performed to improve the normalization of the data when PAHs metabolites concentrations were considered as continuous variables.

### Measurement of plasma IL-9 and eotaxin

Venous blood samples were collected with vacuum blood tube containing EDTA and centrifuged at 3000 g for 10 min. The plasma were isolated and stored in aliquots at −80 °C until use. Levels of IL-9 and eotaxin were measured by using specific ELISA kits (R&D Systems, Minneapolis, MN, USA), according to the manufacturer’s instructions. Each sample was tested in duplicate.

### Statistical analysis

We compared differences of basic characteristics between asthma cases and matched controls using a Chi-square test or Student’s t-test as appropriate, and compared the levels of urinary concentrations of PAHs metabolites using a Wilcoxon signed-rank test. Multiple logistic regression was used to analyze the relationships between urinary PAHs metabolites and asthma, with adjustment for confounding factors such as educational level, occupational dust exposure, family history of asthma, tobacco smoking, pet ownership, flower gardening, physical activity and BMI. Stratified analyses were also performed by gender (male and female), age (<45 years and ≥45 years), BMI (<24 kg/m^2^ and ≥24 kg/m^2^), occupational dust exposure (positive and negative) and tobacco smoking (smoker and nonsmoker). The relationships between PAHs exposure levels and expression of inflammatory cytokines were analyzed by using generalized linear regression, with adjustment for gender, age, occupational dust exposure, tobacco smoking, BMI. The level of significance was set at *P* < 0.05. We performed statistical analyses using SAS software (version 9.1; SAS Institute Inc., USA).
